# Delayed ICU discharges and medical follow-up: a cause of increased mortality?

**DOI:** 10.1186/cc14591

**Published:** 2015-03-16

**Authors:** M Whitaker, MH Spivey

**Affiliations:** 1 Royal Cornwall Hospital, Truro, UK

## Introduction

Discharge from intensive care is a potentially vulnerable time for patients who are recovering from critical illness. Recent data from the ANZICS group have highlighted that the mortality difference in those patients who are discharged out of hours is nearly twice that of those discharged during the day [1]. These results have been replicated in our institution with a mortality of 8.7% (discharged 22:00 to 06:59) versus 4.8% (discharged 07:00 to 21:59). In the UK, NICE CG50 advised that transfer from critical care to the ward out of hours should be avoided and documented as an adverse event. We postulated that one important factor in our hospital is the decreased medical and nursing cover overnight and so looked at the delay from discharge to first medical review and to outreach review.

## Methods

The case notes of 100 consecutive patients discharged to the ward between September 2013 and October 2013 were examined to identify the time of discharge from the ICU and the subsequent first review by the receiving medical team and the Critical Care Outreach team. The grade of the doctor reviewing the patient was recorded.

## Results

**Figure 1:**
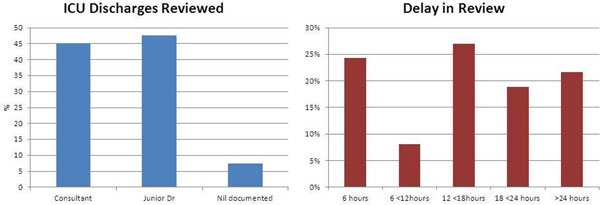


Of these 100 patients, 22 were discharged between 22:00 and 07:59. From the 100 case notes requested, only 50 were available for examination. Forty patients were discharged to the wards, with only 37 having further documented medical reviews in the notes. Only 62% of patients were reviewed by a consultant following intensive care, with over 20% of patients waiting more than 24 hours for any medical review. During this time 18% of patients received a review by the nurse-led outreach team. See Figure 1.

## Conclusion

It is clear that a highly vulnerable group of patients who are recovering from critical illness [2] receive inadequate early follow-up within the hospital. We postulate that the delay in medical review and the lack of senior review may be caused by over 40% being discharged overnight and contribute to the increased mortality seen in our institution and the ANZICS study [1] with nighttime discharges.
